# Sample Entropy as a Tool to Assess Lumbo-Pelvic Movements in a Clinical Test for Low-Back-Pain Patients

**DOI:** 10.3390/e24040437

**Published:** 2022-03-22

**Authors:** Paul Thiry, Olivier Nocent, Fabien Buisseret, William Bertucci, André Thévenon, Emilie Simoneau-Buessinger

**Affiliations:** 1 LAMIH, CNRS, UMR 8201, Université Polytechnique Hauts-de-France, F-59313 Valenciennes, France; emilie.simoneau@uphf.fr; 2 CHU Lille, Université de Lille, F-59000 Lille, France; andre.thevenon@univ-lille.fr; 3 CeREF Technique, Chaussée de Binche 159, 7000 Mons, Belgium; 4 PSMS, Université de Reims Champagne Ardenne, F-51867 Reims, France; olivier.nocent@univ-reims.fr (O.N.); william.bertucci@univ-reims.fr (W.B.); 5 Service de Physique Nucléaire et Subnucléaire, Université de Mons, UMONS Research Institute for Complex Systems, 20 Place du Parc, 7000 Mons, Belgium

**Keywords:** low back pain, sample entropy, regularity, complexity, inertial measurement unit, motion analysis, variability

## Abstract

Low back pain (LBP) obviously reduces the quality of life but is also the world’s leading cause of years lived with disability. Alterations in motor response and changes in movement patterns are expected in LBP patients when compared to healthy people. Such changes in dynamics may be assessed by the nonlinear analysis of kinematical time series recorded from one patient’s motion. Since sample entropy (SampEn) has emerged as a relevant index measuring the complexity of a given time series, we propose the development of a clinical test based on SampEn of a time series recorded by a wearable inertial measurement unit for repeated bending and returns (b and r) of the trunk. Twenty-three healthy participants were asked to perform, in random order, 50 repetitions of this movement by touching a stool and another 50 repetitions by touching a box on the floor. The angular amplitude of the b and r movement and the sample entropy of the three components of the angular velocity and acceleration were computed. We showed that the repetitive b and r “touch the stool” test could indeed be the basis of a clinical test for the evaluation of low-back-pain patients, with an optimal duration of 70 s, acceptable in daily clinical practice.

## 1. Introduction

Low back pain (LBP) is a complex condition with multiple factors contributing to both pain and disability, as well as reduced quality of life [1]. Out of 301 conditions and 188 countries, LBP is the leading cause of years lived with disability [2]. Musculoskeletal conditions account for one third of first-line consultations [3]. On the socio-economic level, considerable direct and indirect costs are generated by acute LBP, which affects nearly 84% of the population at some point in their lives, and especially by chronic non-specific LBP, which has a prevalence of 18.3% to 23% and varies according to economic status, sex, and age [4,5,6,7].

### 1.1. Movement Variations

The presence of persistent chronic pain presumably induces an alteration or adaptation of motor responses in participants with chronic LBP. Developing new diagnostic tools leading to the identification and assessment of these sensory-motor changes is a current challenge in the field, with the aim of eventually building better rehabilitation programmes [8].

Sensorimotor control is inherently variable for each movement in the same individual and from one individual to another [9]. The idea that lumbopelvic movement features change in patients with LBP compared to healthy participants has already been explored in previous works. In [10], it was shown, through a maximum trunk flexion and return test performed three times consecutively with a one-minute rest phase between each repetition, that the displacement of the instantaneous lumbopelvic centre of rotation is smaller and less variable in LBP patients than in healthy subjects and in participants who have recovered from an episode of LBP. This smaller-amplitude and less variable displacement is expected to increase the risk of tissue damage by exposure to some form of overload. The links between variability and overload have also been explored in [11] by resorting to surface EMG. It appears that, in healthy participants, variability in muscle activity avoided overloading an area and allowed for better endurance during repeated tasks [11]. Such variations of muscle activity should have an observable impact on kinematic data, on which we focus in the present study.

A systematic review and meta-analysis of 43 studies analysed and compared lumbopelvic kinematics in participants with and without LBP [12]. The authors concluded that people with LBP tend to have reduced range of motion and speed of movement and poorer proprioception, while healthy participants can adapt to fatigue by performing less stereotyped and more varied lumbar movements that reduce the load on fatigable structures. People without pain show a more complex and less predictable rate of lumbar movement with a lower degree of structure in its variability, whereas people with LBP do not [13]. This last point highlights the two different concepts related to the notion of variability, namely the range of variation (which we call variability) and the complexity of variation (which we call complexity).

### 1.2. Complexity and Entropy

Movement variability is inherent to human motion and our meaning for the concepts of variability and complexity must be further discussed so that the results of a clinical test using these quantities can be understood and interpreted.

In “traditional”, linear, analysis of the movement, one measures average parameters such as speed, frequency (number of repetitions over time) and amplitude. The variability is assessed by calculating the corresponding standard deviations (SD), measuring the magnitude of variability. However, most human movements have nonlinear features, and an exclusive linear modelling may be a source of misinterpretation [14]. It is therefore necessary to move towards nonlinear analysis to assess more subtle (but still related to clinical observations) features of movement such as predictability or complexity. A recent study indicated that pain induces, among other things, a reduction in the degrees of freedom available during natural movements which becomes more evident during more demanding tasks, and concluded that chronic LBP reduces the complexity of movements [15]. Although it can be readily concluded that there is an added value in including non-linear indices as part of an overall statistical protocol, the best index to choose is still a matter of debate [16]. There is nevertheless a consensus about the relevance of sample entropy (SampEn) to assess the complexity of a given time series [17,18,19].

Initially, entropy is a concept that deals with the randomness and predictability of a system, a greater entropy being associated with more randomness and less order in the observed system [16]. Therefore, entropy assesses the complexity of a given time series variability. In a historical, thermodynamical sense, computing entropy would demand an infinite time series. On the contrary, clinical measurements often result in short time series. SampEn is a way of computing entropy reliable for short time series and capable of providing more clinically coherent parameters [16,20,21].

For a test to be feasible in clinical practice, it is important that it is time-limited with a secure amplitude for the patient according to the contingencies of that practice. Taking measurements over a period of one minute or less (optimal duration), with the highest possible accuracy, meets this contingency criterion. Comparing different test amplitudes and durations could therefore be useful. It has been shown that SampEn is independent of the length of the data by remaining conservative under 200 data points [17] and that SampEn shows relative consistency with another way of computing entropy called Approximate Entropy [22,23]. SampEn allows a relevant assessment of the complexity of short time series in human motion [22].

Recent studies have shown that it is not only possible to differentiate LBP patients from healthy participants with an accuracy of 96% but also, via a machine learning system analysing kinematic data from two Inertial Measurement Units (IMU), to separate LBP patients into two main groups, high vs. low-medium risk [24,25,26]. These studies provided a diagnosis based on raw data from the IMU without any physiological explanation, working as a “black box” trained with sample data. We hypothesise that the analysis of the variability of these measurements should provide additional information to better understand and guide the clinical management of LBP patients. More precisely, an LBP patient is expected to show less variability in its movement pattern due to pathology, resulting in a decrease in entropy—this feature is actually expected in general when comparing healthy subjects to patients [14]. In the context of a repeated lumbo-pelvic bending and return (b and r) movement, for a condition such as LBP, we therefore expect SampEn of the angular velocity with the horizontal axis in the sagittal plane to provide clinical information on the complexity of this movement and therefore also on the adaptive capacity of the motor control system. The use of low-cost IMUs and dedicated user-friendly software, nowadays widely affordable, should allow clinicians to have access to kinematic data in the management of LBP [27].

### 1.3. Lumbar Flexion

To develop a physical test that is easy to perform in primary clinical practice, the choice of b and r, and thus movements in the sagittal plane, seems appropriate [10,11,28,29]—it is a movement used in many situations of daily activities. Pelvic movements in the sagittal plane are part of lumbopelvic flexion-extension [28]. The management and follow-up of LBP requires assessment and reassessment of b and r, which is a simple and very common movement in the lumbopelvic region. Currently, there is still a lack of availability and clinical feasibility of tools and techniques, mainly due to the limitation of using equipment and methods designed primarily for research laboratories [24]. There is a real need for easy-to-use and inexpensive tools that can provide recordable data to quantify movement variability in the daily clinical assessment and management of patients with LBP.

The objectives of this study are: (1) to determine the length of the time series needed to assess the variability and complexity of the kinematic data in the sagittal plane recorded by a single IMU; (2) to study the relevance of SampEn in this evaluation; (3) to define the optimal b and r amplitude for using this test in a clinical setting.

## 2. Materials and Methods

### 2.1. Participants

The measurements were taken on 25 healthy volunteers recruited by convenience among the students or teachers of the Haute Ecole de Louvain en Hainaut (HELHa, Montignies-sur-Sambre, Belgium). The recruitment announcement was launched via the HELHa intranet system. The study was validated by the Academic Bioethics Committee (https://www.a-e-c.eu/ (accessed on 22 June 2020)) under the number B200-2020-092.

We recruited participants based on the following inclusion criteria: healthy participants between 18 and 30 years old, belonging to the physiotherapy or occupational therapy section of HELHa. To participate in the study, participants had to sign an informed consent form. We rejected participants with one or more of the following noninclusion criteria: participants with a knee prosthesis, a hip prosthesis or having undergone a spinal operation, participants with any surgical history of less than 12 months, participants with scoliosis or any other spinal deformity, participants with musculoskeletal disorders, participants who have engaged in strenuous physical activity within 24 h prior to the measurement, and participants with any pain experienced during the measurement.

### 2.2. Measurements

**Figure 1:**
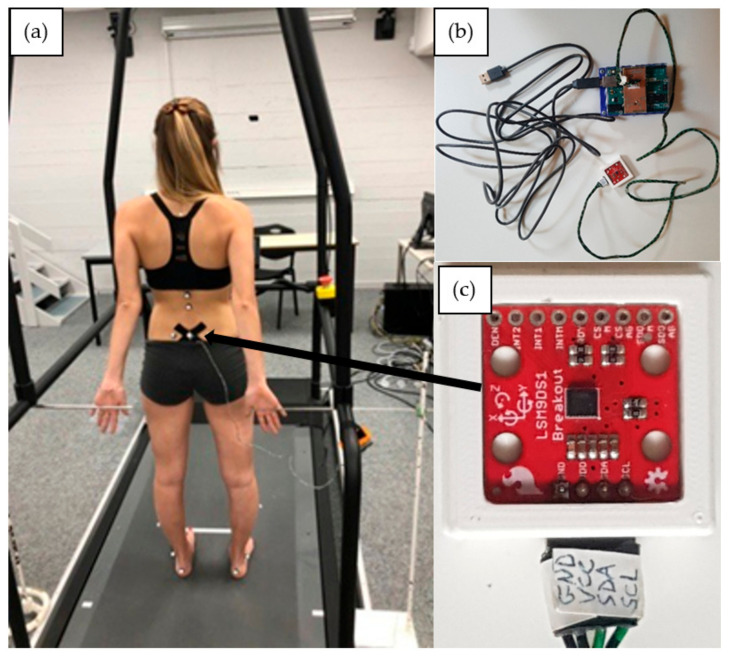
(**a**) Starting position of the participant. The visual cue (black rectangle) is observable on the wall and the metal bars are observable on the left and right sides of the picture. (**b**) The sensor system. The IMU is below, while the controller is above. (**c**) A zoom on the sensor as placed on the participant.

Participants were equipped with a new sensor system called DYSKIMOT, developed to be low-cost; see ref. [30] for a detailed presentation. The DYSKIMOT sensor is a magnetic angular rate and gravity sensor based on the IMU LSM9DS1 micro-electro-mechanical system, with a mass of 10.44 g and a size of 3 × 3 cm (Figure 1c). It consists of a 3-axis accelerometer, gyrometer and magnetometer, as well as a temperature sensor. These internal components measure acceleration (in [g], ±16 [g]), angular speed (in [°/s], ±2000 [°/s]) and magnetic field (in [gauss], ±16 [gauss]), respectively. The device, whose sensitivity depends on the selected range, can operate between −40 and +85 °C. Communication with other electronic components is via a Serial Peripheral Interface bus (SPI) or Integrated Circuit Protocol (I2C). The data, recorded at the maximum achievable sampling rate of 100 Hz, is transmitted to a PC via an Arduino Uno Rev 3 and a USB cable (RS232 serial link) (Figure 1b). The Arduino contains the data retrieval program, using the SparkFun library provided for this sensor, which is then transferred to a dedicated acquisition software [30]. In studies addressing lumbopelvic movements, the sensors were most often positioned at the top of the lumbar spine opposite to the 10th and 11th thoracic vertebra (T10, T11) or to the 1st lumbar vertebra (L1) and at the level of the sacrum on the 1st or 2nd sacral vertebra (S1, S2) and laterally on the right thigh [31,32,33,34]. Some authors also placed the sensor at the xyphoid appendix [34]. In this study, we were interested in pelvic movements only. The DYSKIMOT inertial sensor was therefore positioned on the skin facing S2 with the X-axis pointing to the left, the Y-axis pointing upwards and the Z-axis pointing backwards (Figure 1c).

**Figure 2:**
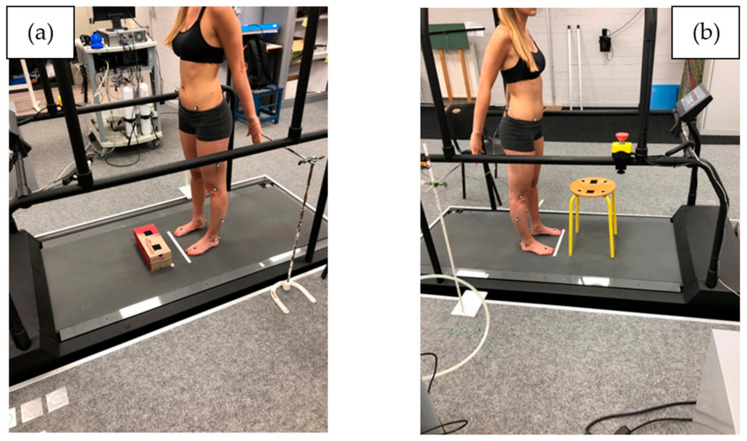
(**a**) Starting position for b and r box test; (**b**) starting position for b and r stool test. Box and stool are placed 10 cm in front of the participant’s toe marker.

The task asked to our participants was much in the spirit of [11], in which healthy participants and chronic LBP patients were asked to move a box (40 × 20 × 30 cm) weighted with a 5 kg weight repeatedly between 2 superimposed shelves during 25 cycles (200 s). The duration of the task was chosen based on pilot tests confirming the patients’ ability to perform the task successfully without having to interrupt it due to pain or excessive fatigue. In our study, participants were asked to perform a series of 50 repetitions of lumbopelvic b and r movements at a comfortable pace. We informed them that they could stop the repetitions if they felt pain. Each participant had to perform the b and r test twice under two randomised conditions. The first condition consisted of touching an 11.4 cm-high cardboard box placed on the floor 10 cm from the participant’s feet, as shown in Figure 2a. The second condition consisted of touching a four-legged stool 46 cm high, also placed 10 cm from the participant’s feet (Figure 2b). On return, the subject was asked to touch with each of their hands each of the metal bars placed behind them. The bars were placed at the height of the hands of each subject in the standing position, 34 cm behind the vertical of the toe marker.

### 2.3. Protocol

Anthropometric characteristics of all participants were first measured before the experiment: age, height, body mass. The posterior-superior iliac spines were located by palpation, and an imaginary line drawn between them allowed the locating of the second sacral vertebra (S2) which is situated in the middle of this line. The sensor was then attached to the participant’s skin with double-sided hypoallergenic adhesive tape.

Before starting the measurement, a visual cue was placed 2 to 4 m in front of the subject at a height of 1.65 m to facilitate the return to the initial position and to ensure that the gaze was horizontal in this same position. The participant stood in the upright position and touched the two horizontal guides placed behind them. The head was in the Frankfurt plane with the gaze directed towards the visual cue placed in front of them. The examiner then gave instructions: “Starting from the initial position, lean forward and touch the two targets on the stool/box. Then stand up and return to the starting position. Repeat the movement 50 times quickly but without rushing and while remaining comfortable for you. All without changing the position of your feet during the test and without talking.”

Ten trial movements were carried out to ensure that the participant had fully integrated the instructions for both tests (box and stool) and to familiarise themselves with the environment. After this test, the participant was given 2 min of rest (sufficient time due to the low intensity of effort for the ten repetitions) before the measurement began. After this rest period, the participant performed the 50 flexion-extension repetitions. Participants were randomly allocated to two groups, one starting with the box and the other starting with the stool. Randomisation was performed via the Random^®^ mobile application (Notrobots, Pordenone, Italy).

**Figure 3:**
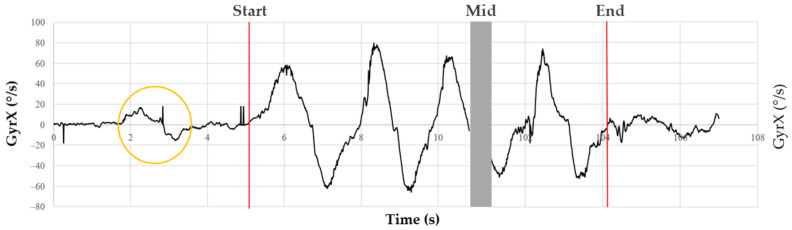
Typical trace of GyrX versus time during b and r movements (4 cycles are displayed). The yellow circle shows the left–right rotation asked before beginning the b and r movements. The first red line shows the closest point to 0 just before the start of the first b and r cycle, and the second red line shows the closest point to 0 just after the last b and r cycle, as it can be easily seen.

The placement and protocol were achieved by the same experimenter. The acceleration and angular velocity time series were recorded on a PC using dedicated data acquisition software developed for the DYSKIMOT sensors (see above). The 6 times series are referred to as AccX, AccY, AccZ (acceleration) and GyrX, GyrY, GyrZ (angular velocity) in the following. Participants were asked to perform a left–right rotation followed by a return to the neutral position just before starting the b and r movements. It allowed a localisation of the start of the 50 b and r movements by visual inspection of the time series. A typical trace of GyrX is shown in Figure 3. The last point of the series was manually selected as the last point closest to 0°/s that ends the last b and r cycle. The typical length of the recorded time series was not far from 2 min (12,000 points), which is much longer than the typical length one would expect in clinical applications. For clinicians, tests should be as time-efficient as possible, and for patients with low back pain, a repeated test should be as short as possible to avoid aggravating symptoms. To meet these conditions of clinical feasibility, such a test should not exceed one minute.

### 2.4. Data Analysis

We first compared the length (duration) of the 6 time series from the box-test to those of the stool-test by using a Wilcoxon signed-rank test. A linear regression of the box-test duration vs. stool-test duration was also performed. Beyond duration, movement amplitude is the most obvious parameter to be measured in clinical practice. It is important to show that the sensor used can compute amplitudes, without needing an extra goniometer. The angular amplitudes of b and r in the sagittal plane were then calculated by integrating GyrX for both tests. The successive minimum (min_i_) and maximum (max_i_) angles were identified, the consecutive amplitudes (A_i_ = max_i_ − min_i_) were computed and the average amplitude *A* was calculated for a given test. A paired *t*-test was made to compare the amplitudes of the box-test to those of the stool-test.

Regarding variability, its magnitude for a periodic time series may be assessed by computing its standard deviation (SD). We calculated the SDs of the angular velocities around the 3 axes for both the b and r box test and the b and r stool test, i.e. SDX, SDY and SDZ. Comparisons between SDX, SDY and SDZ for both tests were performed by a Wilcoxon signed-rank test. Linear regression of the box-test SDs vs. stool-test SDs was also performed. SampEn values were then calculated from the full available time series by using a homemade routine based on the definition given in [17,35], relevant for short time series but longer than 200 points. These values are denoted as SampEn50. Technical details about SampEn are given in Appendix A.

However, 50 repetitions of a b and r movement may be too time-consuming for a clinical test performed in daily practice. The duration of the test must meet the criteria of feasibility and ease of clinical use and those imposed by the minimum length of a time series to obtain a robust SampEn value. To determine this test amplitude and length, we calculated the SampEn by splitting our time series into 2 s increments (200 data at 100 Hz). The first 10 s of measurement were ignored to exclude potential habituation effects, and data after the first 70 s were neglected so that time series with equal lengths were considered (the fastest participant performed the test in about 70 s). An R routine was then programmed to calculate SampEn on partial time series, i.e. from 10 to 12 s, from 10 to 14 s, …, from 10 to 70 s. These partial SampEn values were denoted as SampEn10I, with I = 12, 14, etc. An analysis of the degree of similarity between SampEn50 and the partial SampEn was achieved by Pearson correlations and linear regressions of the form SampEn10I = k SampEn50 between these values for all participants in both b and r tests. Through this analysis, we hoped to determine an optimal test duration for all patients in clinical use, i.e. the SampEn value that was as close as possible to SampEn50 (high precision), but with the shortest possible time series to ensure clinical feasibility as explained above. The value retained for I is the smallest one such that the coefficient of determination R^2^ is above 0.99, with a slope *k* above 0.95. We consider that a degree of linkage better than 95% in the prediction of SampEn is satisfactory in daily clinical use. The 95% levels of agreement of differences between the entropy values (Gyr X) were defined using the method of Bland and Altman [36]. The entropy differences were drawn in relation to the mean values, and 95% of the differences were expected to lie between the 2 limits of agreement. These 2 limits of agreement were the mean differences ± standard deviation expressed as bias ± random error according to Atkinson and Nevill [37].

Computations of the amplitudes, standard deviations, sample entropies, and linear regression parameters were made with R free software (v. 4.1.0). Statistical tests were performed by using Sigmaplot (v. 14.0, Systat Software, San Jose, CA, USA) with a 5% significance level.

## 3. Results

### 3.1. Population

Here, 25 participants were selected. One was excluded because of scoliosis. The data of a second participant could not be analysed, due to the incomplete recording of data by the DYSKIMOT software during the test. This brought the total number of participants to 23; the population’s features are shown in Table 1.

### 3.2. Duration and Amplitude

**Figure 4:**
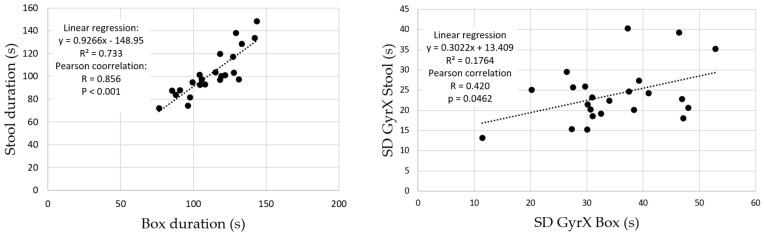
Correlation coefficient and linear regression for duration and SDs (amplitude, e.g., SDX) for the 50 b and r cycles.

The duration and SDs (amplitude) of the 50 cycles of the b and r movement changed significantly between the two tests (*p* < 0.001 to *p* = 0.002), see Table 2. The correlations and the linear regression are presented in Figure 4.

We note that both tests lasted at least 72 s, which justifies our choice to truncate both time series from 10 to 70 s for the calculation of partial SampEn.

### 3.3. SampEn and Optimal Duration

SampEn50 values are displayed for both tests and for the 6 time series in Table 3. The minimal (maximal) value was obtained for GyrX (AccX) regardless of the test.

There was no correlation between the SampEn values of the two b and r tests. All Pearson correlation coefficients were around or below 0.5. The best (but insufficient) correlation (but insufficient) coefficient was for AccZ with R = 0.559 with *p* < 0.01.

**Figure 5:**
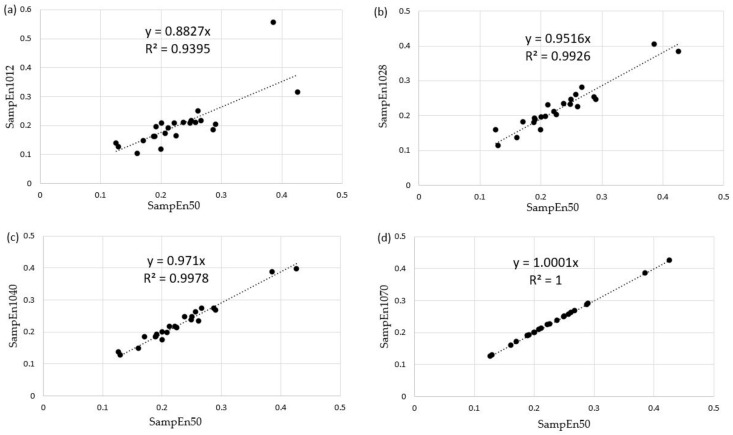
Typical linear regressions for SampEn50 vs. SampEn10I computed for GyrX in the b and r stool test; the regression was performed on the 23 participants (black points). (**a**) SampEn50 vs. SampEn1012. (**b**) SampEn50 vs. SampEn1028. (**c**) SampEn50 vs. SampEn1040. (**d**) SampEn50 vs. SampEn1070.

**Figure 6:**
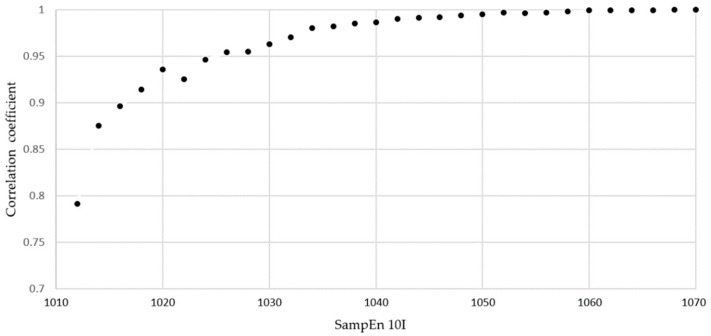
Evolution of the correlation coefficients between SampEn 50 and SampEn 10I for GyrX in the b and r stool test. All corresponding *p*-values are <0.01.

Typical linear regressions of SampEn50 vs. SampEn10I are shown in Figure 5. The evolution of the correlation coefficient GyrX-stool test is shown in Figure 6.

## 4. Discussion

Variability in sensorimotor control should be considered as normal and useful; its reduction or increase seems to be related to pathology. To quote [38]: “Far from being a source of error, the evidence supports the need for an optimal state of variability for health and functional movement”. The variability in healthy individuals shows a temporal organisation characterised by a chaotic behaviour that allows the system to be optimally adaptable. By reducing or increasing its capacity to vary, a biological system loses its adaptability or stability [38]. This paradigm is exemplified by the results of [39] stating that, within the possible spectrum of motor control adaptations to pain in LBP patients, two main phenotypes seem to emerge: increased control or lost control involving, via different mechanisms, abnormal tissue loading in the lumbar region.

Many joint levels are involved in b and r movement, such as dorsal and lumbar flexion-extension, pelvic tilt, flexion-extension of the hip, knee, and ankle. In the case of b and r, pelvic tilt is related to the amplitude ratio of flexion-extension of the hip, knee, ankle, and dorsal-lumbar region. A limitation induced by using a single IMU is that movements other than those of the trunk cannot be studied. For example, the foot and pelvic position influence each other [28]. The stage of flexion-extension influences the level of synchronisation between the pelvis and the spine during b and r with greater involvement of the pelvis during the final and initial phases of forward flexion and backward extension, respectively [39]. Speed is also an important factor that can alter movement strategies to adapt functionally to reduce stress on structures and avoid pain when LBP patients change to optimal complexity and adopt a stereotyped lumbopelvic rhythm [40,41,42].

The use of a sensor allows information to be obtained that goes beyond amplitude: by recording angular velocity and acceleration, for example, one has access to motor strategies used to perform the whole motion. In particular, the calculation of SampEn may assess the complexity of these time series.

Although there is a significant difference between the execution time of the two b and r tests, these two durations are well correlated with a regression slope approaching 1. This implies a very small difference in duration between the two tests always in favour of the box test. Therefore, the execution speeds are higher in the box test than in the stool test. This higher speed could be an obstacle to the performance of the test in clinical practice. It was shown in [38] that in a flexion task performed at different speed levels, the asymptomatic group moved with a progressively higher degree of lumbopelvic coordination as speed and acceleration increased, whereas the LBP group adopted a uniform lumbopelvic pattern for all speed levels examined. There is therefore no point in increasing the speed of execution in a clinical test regarding LBP. This suggests that the stool test should be preferred in clinical practice. Indeed, the stool test has a significantly lower angular amplitude for a similar although significantly different duration, resulting in lower angular velocities and therefore less physical strain for the participant performing the test. This reinforces the choice by reducing the risk of worsening clinical symptoms and signs in the patient being tested. However, care should be taken in the use of either test (box and stool) in future research due to their tendency to be significantly different in the distribution of SD and SampEn50 values.

As the b and r task takes place mainly in the sagittal plane, the angular velocity around the transverse axis (X), as mentioned above, shows a greater regularity compared to those around the other two axes. This more structured pattern leads to a lower value of SampEn50. It is correlated with the fact that AccZ has the lowest entropy amongst the accelerations: as seen from the sensor frame, a rotation of the X-axis induces a centripetal acceleration along the Z-axis. The reason for these lower SampEn values is that both GyrX and AccZ time series contain kinematic information about the b and r rotations performed, i.e. the target to touch on the box or stool. This tends to confirm the hypothesis already put forward, according to which an individual develops a movement strategy aimed at reducing the complexity to increase the precision of a targeted movement during a task [42]. Along these lines, it can be concluded that the large Sampen50 value for AccX stands for the fact that no planned linear movement occurs along the X-axis. Note that SD and SampEn show opposite behaviours when comparing the different directions: this tends to confirm the different descriptive role played by these two analysis tools.

**Figure 7:**
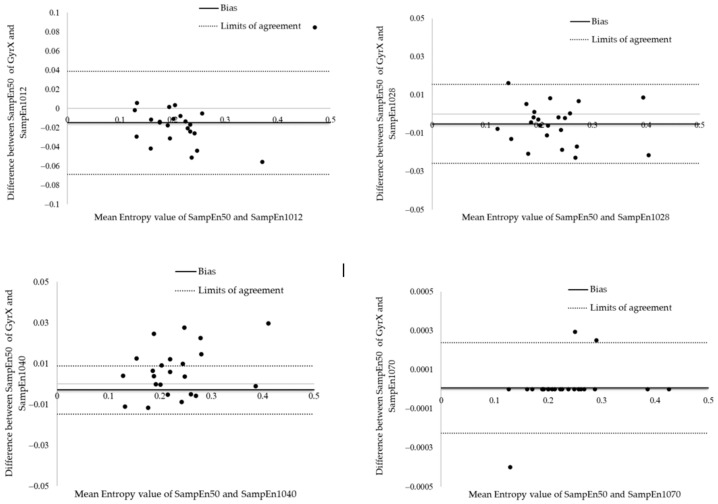
Bland and Altman plots illustrating the differences between SampEn 50 and SampEn 10I for GyrX in the b and r stool test.

The duration of a clinical test involving repeated movements should not be too long, to obtain and maintain patient compliance. As shown above, a 70 s measurement including a 10 s habituation phase and a 60 s actual test phase yields SampEn values for the three-axis accelerometer and three-axis gyroscope that are almost perfectly equivalent to those that would have been measured over 50 repetitions in the b and r test (R^2^ and slope equal to 1 up to software precision). However, satisfactory SampEn values (R^2^ = 0.99926 and slope of 0.9516) were already obtained after 18 s of testing. The Bland and Altman analysis shown in Figure 7 reveal that the mean bias between SampEn50 and SampEn1028 was −0.0052 ± 0.0105 with 95% limits of agreement of the bias from −0.0259 to 0.0154. These results suggest a great agreement between the SampEn50 and SampEn1028 values. For future research, a measurement over 60 s with a 10 s warm-up should be considered, although measurements over 20 s are already satisfactory in daily practice.

## 5. Conclusions and Outlook

A b and r stool test performed over a period ranging from 20 to 60 s with a warm-up time of 10 s with a single inertial sensor placed in the low back seems to be relevant for the evaluation of low back kinematics complexity through a SampEn computation. The next step towards the development of the test will be the analysis of the inter- and intra-examiner reliability of the sample entropies obtained in the b and r stool test.

Then, we will have to prove the clinical validity of these values in the evaluation of low back pain by verifying that a low-back-pain population indeed has a significantly different sample entropy than a healthy or recovered population. The more stereotyped response of the lumbar-pelvic region observed in patients with low back pain compared to healthy participants points towards such a result [40].

Finally, automation of SampEn calculations should be introduced into the data acquisition software to allow for clinical use. Such a work is in progress.

## Figures and Tables

**Table 1:** Anthropometric data for our population of 23 healthy participants. Continuous variables are displayed under the form mean ± SD.

Parameter	Mean
Male/Female	10/13
Age (year)	22.5 ± 2.5
Height (m)	1.72 ± 0.1
Body mass (kg)	66.8 ± 10.3
BMI (kg/m^2^)	22.6 ± 2.5

**Table 2:** Duration and SDs for the 50 b and r cycles. *p*-values from Wilcoxon signed-rank tests (duration and SDs) are shown (box versus stool).

Test	Min Duration (s)	Max Duration (s)		Mean (s)	MED	Q1	Q3	*p*-Value
box	76.3	143.3		112.2 ± 18.5	11.5	9.8	12.8	<0.001
stool	72.3	148.4		102.5 ± 20.0	9.7	8.8	11.7	
Test	SDX (°/s)	*p*-value	SDY (°/s)	*p*-value	SDZ (°/s)			*p*-value
box	34.6 ± 9.7	<0.001	7.5 ± 2.4	0.002	7.4 ± 2.6			0.002
stool	23.9 ± 7		6.3 ± 2.4		6.2 ± 1.8			

**Table 3:** Values of SampEn50 for the 6 available time series for both b and r tests and *p*-values of Wilcoxon signed rank box-test vs. Stool-test.

**Test**	**GyrX**	***p*-Value**	**GyrY**	***p*-Value**	**GyrZ**	***p*-Value**
box	0.218 ± 0.106	0.149	0.639 ± 0.083	0.01	0.646 ± 0.108	0.01
stool	0.232 ± 0.071		0.607 ± 0.088		0.612 ± 0.073	
**Test**	**AccX**	***p*-value**	AccY	***p*-value**	**AccZ**	***p*-value**
box	1.060 ± 0.326	0.796	0.567 ± 0.235	0.988	0.233 ± 0.087	<0.001
stool	1.026 ± 0.305		0.578 ± 0.192		0.331 ± 0.125	

**Figure A1:**
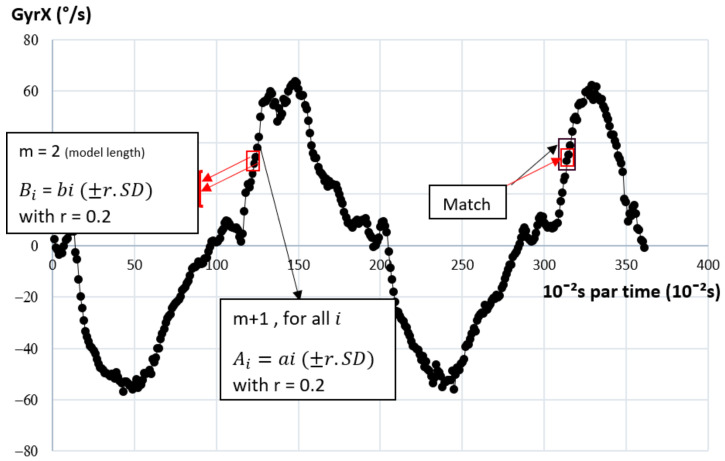
Schematic representation of SampEn calculation from a typical GyrX time series, adapted from [43]. The value *m* = 2 is chosen.

## Data Availability

Data are available at https://osf.io/t4dgr/ (18 January 2022).

## Appendix A. More about SampEn

We present in Figure A1 a schematic presentation of the SampEn estimation method on a sample angular velocity time series freely inspired from [43]. One starts by considering a template vector of length *m* beginning at instant *i.* Then, one counts the number of pairs, A_*m*_, of vectors of length *m* that are “close enough” to the template vector. The criterion is defined through a given norm (Euclidean distance in our case), and the distance *d* below which two vectors are regarded as close enough is conventionally expressed in the fraction of the times series standard deviation *SD*, i.e. *d* < *r SD* with *r* < 1. The same calculation may be performed by extending the template vector to a length *m* + 1, leading to B_*m*_. Sample entropy is then defined as [43] $\mathrm{SampEn}=-\ln\frac{A_{m}}{B_{m}}$. Note that the search for pairs of vectors is performed within a window of N points, which is another parameter of the calculation.
